# Monthly Summary

**Published:** 1892-07

**Authors:** 


### Monthly Summary.

THE NEWEST DISINFECTANTS FOR ROOT CANALS.—By Dr. All. Bandmann, of Berlin.—Extract from a lecture before the "American Dental Club, of Berlin."—As you are aware the aniline coloring substances have been repeatedly and successfully used in medicine as disinfectants for about two years. I have used two of these substances in my practice for several months, and have found them successful.

The coloring matters, of which I shall treat, are pyoctanin and methylen-blue, especially in their nature as antiseptics. I have used them in three different applications, *viz.*, first, fistula; second, decayed pulp; and third, alveolar necrose. I have first employed the pyoctanin, but then turned to methylen-blue, for I have achieved with the latter far quicker results.

In fistula, I have used the following method: After having secured a good, wide entrance to the root canal, I have injected, by means of a syringe, a drop of methylen-blue, having before surrounded the tooth with cotton rolls, for the methylen-blue colors very intensely, and then tightly closed the cavity with a large cotton stopper.

For decayed pulp, I have used the methylen blue as a disinfectant, by dropping cotton in a solution of methylen blue, and introducing it into the canal by means of a probe, then closing, as above. Lately, I have dipped the cotton also in methylen-blue powder. By severe beating, all these particles of powder that have not become damp, fall away, and then I introduce the cotton. In this way, I have removed from the canal every odor in a considerable shorter time.

For the alveolar necrose I have first used methylen-blue, after removing the sequestrum and scrubbing away the disease germs. I was surprised to see in what short time the wound healed; but I am convinced that the alveolar necrose can be treated safely and rapidly from the very first stage with methylen-blue.—*Journal Jur Zahnheilkunde.*

THE MEDICAL LAW AS IT CONCERNS DOCTORS NOW PRACTICING.—The new law relates only to persons who shall after the date of its passage begin the practice of medicine within the State. In regard to any person who may have previously practiced in Maryland, it is silent. There persons occupy after its passage exactly the legal status which they occupied before. The new boards of examiners are not expected to indorse their claims as practitioners, nor to condemn them as in any way culpable. They are simply to ignore them, and to confine their attention to future practitioners.

Moreover, the new Boards of Examiners may not molest any person hereafter beginning practice in Maryland, unless they have before them reasonable proof that such persons have never, previous to the passage of the law, practiced medicine in any other of the United States.

It will appear, therefore, that considerable caution must be exercised in the arrest of practitioners who hail from other States. Many years will pass before the law will be of much use in keeping itinerant doctors out of Maryland.

A good beginning has, however, been made, and its beneficent influence in restraining young graduates of home colleges will immediately be felt—*Md. Med. Journal.*

---

ARISTOL.—Modern surgery has been fortunate in many of the newer agents it has added to its resources, and among these aristol must be cited as the most notable. It is difficult to realize, to-day, that previously to the discovery of aristol there was hardly a general laboratory in America or Europe in which experimenters were not constantly employed in seeking for a suitable substitute for iodoform, while it was hardly possible to keep up a medical journal without reading of some new (and always inadequate) method of masking the awful odor of that surgical abomination. Now we rarely meet with these recipes, and all surgeons know the value of aristol. Surgery waited long for this topical remedy, but its splendid qualities redeemed its tardiness. The absence of offensive and toxic properties united in its healing qualities were fully sufficient of themselves to make a great reputation for aristol. But its value as a dressing was still more highly appreciated when it found that it would adhere closely to denuded surfaces and make an impervious coating over them. Ulcerated areas which had defied the classical applications quickly closed under the stimulating influence of aristol, and in many gynæcological cases in which iodoform was inadmissable aristol did the work promptly and effectually. In burns, scalds, bedsores and as a dressing for blisters, aristol is the most eligible application, and it is indicated in all of the operations of minor surgery. It has been successfully used, also, in diseased conditions of the eye, ear and nose, while in rectal surgery it is recognized as having special value. In major operations its superiority over iodoform is specially marked on account of its freedom from toxic influences; and in abdominal surgery aristol has no superior. Dental surgeons, also, have given it their unqualified approval. Indeed, the range of aristol has become so widened that it is regarded rather as the superior of than a substitute for that once useful but malodorous remedy, iodoform.—*Md. Medical Journal.*

---

ARREST AND TRIAL OF A TRAVELING MEDICAL MAN.—Considerable interest was excited by the case of the State of Maryland vs. Charles McKay, alias Oregon Charley, which took up the better part of Friday, June 17th, in its trial before Justice Bitner.

The traverser in the case has for some time past had a tent pitched in the neighboreood of Locust and Bethel Streets, in this city, where he has been consulted by many persons for relief from various bodily troubles and ailments, has given them advice, furnished medicine, etc. He appeared to be doing well and to be giving general satisfaction to those that consulted with him and followed his advice.

Friday he was brought before the Justice charged with practicing medicine in violation of the laws of the State.

The law under which he was accused and tried was passed at the last session of the Maryland Legislature and has not yet been published in book form, the only evidence of the law being a written copy certified to by the Clerk of the Court of Appeals.

The law provides, under penalties ranging from fifty to two hundred dollars, that no one who is not now a practitioner of medicine shall be allowed to practice in this State, without possessing a diploma from some regular medical college, on obtaining a license to do so, either from the State Board of Regular physicians or the Board of Homœopathic physicians, which are the only two medical schools that are recognized under the new law.

Although the traverser had no such license to practice in Maryland, and produced no diploma from a medical college, yet there was no evidence to show that the traverser had not practiced medicine outside the State of Maryland. and as the law was entirely prospective in its operations and referred to those who were not practitioners at the time of its passage, the magistrate rendered a judgment of "not guilty."

If the party came to this State after the passage of the Act under which he had been arrested, yet even then, if he had been a practitioner of medicine in another State, the Constitution of the United States would protect him in the exercise of his profession here, as that instrument clearly prohibits any State from passing a law interfering with the rights and privileges of a citizen of any other State. It was incumbent upon the State to show that the traverser had not been a practitioner in the State from which he came, and this they had totally failed to do.—*Hagerstown Daily News.*

---

COURTS DIFFER IN REGARD TO INHALING GAS.—The United States Circuit Court for Illinois differs with the Court of Appeals of this State on the question of the liability of accident insurance companies for death by inhaling gas.

In a recent case in Chicago, a person was found dead at a hotel with the gas turned on in the room. There was no visible sign of violence or external injury on the body, and nothing to show whether it was a case of suicide or an accident. The limbs and features were not distorted, and the victim was lying naturally on his side in bed. He held a policy of accident insurance in the Travelers' Insurance Co., which expressly relieved the company from all liability when death occurred by inhaling gas. The court held that the company need not pay the policy, and quoted the adverse opinion in a case in the New York Court of Appeals, where it was said: "In expressing the intention not to be liable for death from inhaling gas, can only be understood to mean a voluntary and intelligent act by the insured, and not an involuntary and unconscious act. Read in that sense and in the light of the context, these words may be interpreted as having reference to medical or surgical treatment in which *ex vi termini* would be included the dentists' work or a suicidal purpose."

The Illinois court comments on this decision and says: "The reasoning by which that court reached its conclusion is not satisfactory to my mind. The language of the policy is so clear as not to require any construction. The words are unequivocal that the defendant does not insure against death caused by inhaling gas.

There is nothing in the terms of the policy intimating or suggesting that the inhalation of gas must be voluntary or involuntary in order to exempt defendant from liability. That the defendant had the right to so limit its liability, there can be no doubt."—*Buffalo Med. and Surg. Journal.*

---

GOLD CROWNS.—Those who would put on a gold crown quickly and well may succeed by following these directions: Take a piece of thin wire (not too thin), and then, with a pair of pliers, twist it round the stump to be operated on, then slip it carefully off and paint it black; now take a piece of stick of some hard wood, something like a rivetting hammer handle; selecting the stick according to the size of the band you require, put it in the vise now while the paint is wet on your shaped wire ring, describing the exact shape of the stump; place it on the top of the stick you have in the vise, and give it one sharp blow with the flat end of a good-sized hammer; take away your wire; now shape your piece of wood carefully and accurately, as the dark mark guides you, till the whole of the dark line disappears; mind you are inside the line, but do not go too far; now make a ferrule of coin gold for the top of your stick, polish and finish on the stick, then take it off and try it on the stump, and you find it a little too small to go on; take a sand-paper disk. and with the engine rough it all round and make it fit on "slick;" this being done, place your ferrule again on the stick, and make another ferrule to fit over it, as you would proceed to make a lid to a box. Now you have a telescope joint. Take off your second ferrule, or collar, and fix on the stump with osteophosphate, having arranged your gold crown to the articulation with strong wax, remove it, and solder your second collar on the crown, and polish; fill your gold crown with copper stopping, and then, as a lid fits on a box, your patient bites the crown into its place, and remove it if you can. In fitting porcelain teeth for the front, in this way, the great trouble will be in getting the pin in the proper position. Drill out the nerve canal, proceed as before, when you have your first collar on, fit your pivot *in situ*, and cut a small piece of platinum gauze the shape of the collar, and drop it inside, touching the pin; force in some resin wax, and bring out the gauze adhering to the pin; now set it in plaster and powdered pumice, and flush some solder over to fix the pin, then a piece of gold plate; having made your second collar, solder the gauze now filled in with plate, and solder to collar No. 2; on this arrange your porcelain tooth in the mouth, and solder it to second collar; your first collar now being fitted to the stump, press it all into place with good cement. A gold crown will be the result that is strong, substantial and durable.—*A. King in Ohio Journal.*

THE ODONTOBLASTS.—I cannot but think that the odontoblast is a rather hardly used cell; it has not only had to persist during the whole active life of the tooth, but has had to form the soft fibril, the investing tubule, and the matrix of dentine, and, in addition to all this, is considered by many to act as a nerve-ending cell, and conduct sensation to the dentine. This latter function is hardly, I think, consistent with its other offices, and with what we know of the action of cells in other parts of the body. This anomaly struck Mr. Hopewell Smith, in a paper on development, to which I have referred elsewhere, and he would rather consider the function of the odontoblast to be wholly sensory, the cell really being a nerve-end organ, and compared by him to the ganglion cells of the spinal cord, other cells than the odontoblasts being concerned in the formation of dentine. In this case, however, one would expect to find very considerable nerve filaments entering the odontoblasts, and it is also remarkable that in old teeth, the odontoblast layer appears to be absent. Professor Kolliker considers the growth of dentine to be partly by conversion and partly by secretion, and that while the central part of the cell persists as the fibril and tubule, the matrix is a secretion from the odontoblasts and other cells of the pulp. Baume holds that the calcification of the dentine takes place in a material secreted by the odontoblasts.—*Howard Mumery in British Journal.*

---

FOR THE TEETH.—Every substance which is pulverized in order to enter into the composition of a dentifrice should be powdered so extremely fine as to be mere dust. Thus pulverized, most substance likely to be used are harmless; but the best and safest basis for any tooth powder is prepared chalk and charcoal, one to three, the charcoal always to be kept tightly shut from the action of the air. Where there is bleeding of the gums, a little borax may be added where there is foulness, some cinchona bark: where whiteness is desired, some common salt. If a little rouge is added to disguise any portion of the powder not rinsed away, it is permissible; but it is better to make the rinsing thorough. Where the teeth are inclined to be loose in their settings, a wash of the tincture of myrrh is efficacious in fixing them more firmly. Many add to their pure chalk and charcoal a trifle of powdered castile soap as a detergent, with camphor for sweetening the breath where there is decay; it being remembered, however, that camphor can only be reduced to powder by grinding it with a pestle after being lightly sprinkled with rectified spirits of wine. Meanwhile, without taking so much trouble, some pulverized orris root, where the teeth are not decaying will usually make the breath as sweet as the violets whose scent it imitates. A very pleasant and useful wash, where there are tender gums, is made by putting half an ounce of dried rose leaves to steep in a gill of boiling water, pressed and strained after three hours, and added to about six ounces of clarified honey, and evaporated for a few hours. This added, when used, to half as much water makes a valuable astringent wash and gargle. An exeellent deodorizer is made by taking equal portions of ground coffee, chocolate and white sugar, with two-thirds as much pulverized charcoal and vanilla, added to just enough dissolved gum-tragacanth to make a stiff gum; bits of this kept in the mouth are said to make a bad breath pleasant.

Another breath-sweetener may be made at home by taking an ounce of extract of liquorice as thick as paste, a third as much powdered catechu, the same of powdered sugar, a sixth as much gum-tragacanth, a third of a fluid drachm of oil of cloves, half as much oil of cassia, and three drops each of oil of nutmeg and essence of ambergris, mixing well together, adding a trifle of rose-water and rolling the whole into tiny globules between the thumb and finger. Then if the little globules are laid on a leaf of silver, and another leaf is placed over them, the whole covered with a half sheet of paper, and rolled lightly beneath the flat open palm for half a minute, as fine and sweet silvered cachous will be the result as can be furnished by the best pharmacist. Take it all in all, care of the teeth *pays*, in comfort, in beauty, in the conservation of health from youth to old age.—*Harper's Weekly.*

---

DEVITALIZING PULPS.—Dr. Miller contributes to this number an excellent paper upon the devitalization of pulps. It will be seen that he depends upon cocaine for obtunding sensibility at the outset. His strictures upon the slovenly methods of many operators, who thrust in a preparation of arsenious acid and then proceed to ram it down upon the irritated pulp with a sandarac wad, are very just. His method of using zinc sulphate as a protective covering is new to us, and will commend itself to every dentist who values the comfort of his patients.—*Dent. Pract. and Advertiser.*

